# Effect of Changes in Canine Thyroid Cancer Terminology on Caregiver Anxiety Levels and Treatment Preferences in a Scenario‐Based Study

**DOI:** 10.1111/vco.13044

**Published:** 2025-02-24

**Authors:** Bryanna M. Glasspool, Laura Blackwood, Kelly L. Bowlt Blacklock

**Affiliations:** ^1^ Hospital for Small Animals, Royal (Dick) School of Veterinary Studies, University of Edinburgh Edinburgh UK

**Keywords:** anxiety, dog, terminology, therapeutics, thyroid cancer

## Abstract

In human medicine, the choice of medical terminology influences patients' choice of management options and associated anxiety levels in relation to their diagnoses. The objective of this study was to determine the association between canine caregiver's treatment choices and anxiety levels when papillary thyroid cancer is described with or without the term *cancer.* This randomised cross‐sectional study surveyed 683 people over 18 years old over 8.5 months. Respondents ranked their treatment preference (total thyroidectomy, active surveillance, medical therapy, or radiation therapy) following a scenario‐based diagnosis of papillary thyroid cancer (PTC), thyroid papillary lesion (TPL) or abnormal cells (AC) in their canine pet. Respondents stated their level of anxiety associated with the diagnosis and treatment choice. Of 683 respondents, 622 (91.7%) were female. When presented with a diagnosis of PTC, TPL or AC, 78.1%, 34.2% and 59.3% of participants, respectively, reported being anxious or very anxious about this diagnosis (*p* < 0.01). Surgery was chosen as a first‐choice treatment for PTC, TPL and AC by 71.8%, 39.8% and 53.8% of respondents, respectively, whereas active surveillance was chosen as a first‐choice treatment by 24.5%, 57.5% and 43.9% of respondents, respectively. There was a statistically significant difference in first‐choice treatment selection (*p* < 0.01) and anxiety levels related to treatment (*p* < 0.01) between the three different terms. The terminology used when presenting caregivers with a diagnosis of PTC influences treatment choices and levels of anxiety.

**Abbreviations**
AC: abnormal cells
PTC: papillary thyroid cancer
TPL: thyroid papillary lesion

## 1 Introduction

The most common canine endocrine tumour occurs in the thyroid gland [1], representing 1%–4% of all canine neoplasms [2, 3]. Thyroid carcinomas are generally categorised into follicular and medullary types [4]. Follicular carcinomas can be further classified (based on histological patterns) as: follicular, papillary, compact (solid), follicular‐compact or anaplastic [1, 4]. In humans, the follicular and papillary subtypes are commonly referred to as differentiated thyroid carcinomas, with papillary carcinoma accounting for approximately 85% of cases [5]. In contrast, in dogs, follicular carcinoma is the most prevalent form of differentiated thyroid carcinoma, while the papillary subtype is rarely observed [6, 7, 8].

Despite differences in the frequency of subtypes of thyroid carcinoma between humans and dogs, the canine thyroid carcinoma model holds significant potential for understanding human disease. Canine and human thyroid tumours often show similar biological behaviours, such as bilateral occurrence and non‐functionality in many patients [9, 10]. However, dogs have a higher incidence of carcinoma and metastasis compared to humans [3, 11]. The canine model therefore provides valuable insight into more aggressive disease courses and metastasis, as thyroid carcinomas in dogs tend to metastasise at a much higher rate than in humans (71% compared to 2%) [12, 13]. These parallels in tumour behaviour, coupled with the availability of similar treatment modalities, make the dog a valuable model for investigating the progression and therapeutic response of thyroid cancer, despite subtype differences. In addition to a practical model of disease, translational oncology offers an opportunity to understand conversations around treatment decision‐making, quality of life and caregiver/patient anxiety. Such conversations may potentially differ between species, and where the interlocutor is the caregiver rather than the patient, for example in veterinary oncology, or where the caregiver is a surrogate for an incapacitated patient. Ozdemir et al. (2021) found that the treatment preferences of human patients and their caregivers were discordant, with patients' primary interest being in extending life, whereas caregivers wanted to balance the quality of life with the length of time extended [14]. This serves as one crucial difference between human caregivers and patients with veterinary caregivers and animal patients; in veterinary medicine, patients can offer no insight into their willingness to pursue treatment.

Several studies exist in human healthcare looking at the effect of medical terminology and presentation of diagnoses on patient treatment preferences [15, 16, 17]. In these papers, using more complicated terminology, including the use of medical terminology rather than lay terms and utilising the term ‘cancer’ at the point of diagnosis, has been found to create higher levels of anxiety; the negative emotional impact of making treatment decisions for others is also recognised in human medicine [18]. Terminology choice can increase the desire for overtreatment of even benign or low‐risk conditions. For example, in humans, papillary thyroid cancer (PTC) represents the most indolent histological subtype [19]. Increased access to health services and imaging advancements in the last few decades have greatly increased the incidence of PTC diagnosis [20] especially in high‐income countries [21]. While an increase in incidence without an accompanying increase in mortality supports the benefits of early treatment, a three‐to four‐fold increase in thyroidectomies as a treatment for those PTC diagnosed in humans suggests that overtreatment of PTC in humans may reflect overtreatment of PTC [22].

By contrast, no such studies exist in veterinary medicine. The objective of this randomised cross‐sectional study was to investigate the effect of terminology on caregivers' anxiety levels and treatment preferences when presented with a diagnosis of PTC for their canine pet. Despite a low prevalence of PTC in dogs as compared to humans, PTC was chosen as a model for diagnostic terminology anxiety over other more prevalent cancers in canine patients due to the ability to compare owner anxiety in our study with patient anxiety in the study by Nickel et al. [20]. This comparison allowed us to examine the effect of diagnostic terminology in human and canine patients, which is potentially relevant when defining clinical models of disease or a patient care pathways.

We hypothesised that our findings would mirror those seen in human patients: the term PTC would lead to higher levels of anxiety and selection of more invasive treatment options when compared with the use of the terms thyroid papillary lesion (TPL) or abnormal cells (AC).

## 2 Methods

This study was reviewed and approved by [committee name withheld for review] (approval number withheld for review).

### 2.1 Study Design

Using a framework developed in studies by Omar et al. [23] and Nickel et al. [20], we designed a randomised cross‐sectional, anonymised survey tailored to caregivers of canine patients (Appendix S1). The study and survey were approved by the [committee name withheld for review] (approval number withheld for review). We distributed the survey online using the Jisc Online Surveys platform between March 6 and November 24, 2022. Links to the platform were shared by 170 global online social media groups with a focus on pets and pet ownership, animal healthcare and veterinary oncology. Participants in the study were over 18 years of age. This was an open survey, resulting in a convenience sample. Participation was voluntary, and there was no preselection of participants. No incentives were offered. All participants and social media groups consented to participation in this study. The client consent form (Appendix S1) was accessed directly online by participants and included information regarding the suggested time to complete the survey, methods of data storage, ethical approval and researcher contact details. After consent was given, participants proceeded directly to the study. Participants had the ability to review and change answers by using a ‘Back’ button. Surveys could only be submitted for analysis if all ‘required’ questions were answered (Appendix S1). Cookies were used to provide unique identifiers, but as no identifying information was collected from participants, completion rates could not be analysed. There was no time limit for questionnaire submission after starting participation.

Our survey presented participants with a scenario whereby their canine pet was assessed by their veterinary surgeon for a cervical mass. The diagnosis provided was either PTC, TPL or AC. Next, we provided participants with information pertaining to five treatment options (total thyroidectomy, active surveillance, medical therapy, radiation therapy or no treatment) and outcomes associated with each treatment option (Table 1). Participants were asked to rank their treatment choices following diagnosis, and using a 5‐point Likert scale, grade their anxiety levels (from not at all anxious to very anxious) relating to the diagnosis (termed ‘diagnosis anxiety’) and treatment choice (termed ‘treatment anxiety’).

**TABLE 1:** Treatment options and associated side effects, complications and risks.

Treatment type	Information provided
Surgery	Removing the entire thyroid gland by surgery, which is potentially curative. With surgery, there is a very small risk of dying from the surgery: less than 1.9%. Complications following surgery are seen in just under 13% of dogs and include bleeding (7.7% of dogs) and pneumonia (3.2% of dogs) [24]. Following surgery, there is a very small risk of low thyroid levels which necessitates lifelong medication (one tablet 1‐2x daily) [25].
Monitoring (active surveillance):	Lifelong monitoring of the thyroid for growth or spread.
Medical therapy (e.g., chemotherapy):	Chemotherapy is provided via a series of weekly treatments into the patient's vein and has not classically been used in the treatment of thyroid cancer. Of dogs treated with chemotherapy and depending on the drug used, 20%–50% will show some response [26].However, because chemotherapy is not commonly used, its role in extending survival time has not been evaluated. Indications for chemotherapy may include cancer which is recurrent, which has spread or which is extremely large and not amenable to surgery. Potential complications should be minimal but include vomiting/diarrhoea and an increased risk of developing infections [27].
Radiation therapy:	Radiation is more commonly indicated for very large thyroid tumours which are not amenable to surgery. Treatment consists of 4 weekly treatments and mean survival time is around 45 months. Potential side effects are generally reversible and include hair loss and skin irritation over the neck. Permanent side effects include changes in hair and skin colour over the radiation site [26].

We considered a randomised crossover study to be too lengthy for participants to fully engage with it [20, 23]. Therefore, each respondent was only presented with a single scenario. Scenarios were assigned based on the respondent's month of birth: those born January–April, May–August and September–December were allocated the diagnosis of PTC, ACs and TPL, respectively.

Finally, at the end of the survey, we collected anonymised data pertaining to respondent demographics, including self‐identified gender, age, highest completed level of education, pet ownership and experience with thyroid masses (lumps) or cancer diagnoses in their pet or family. Surveys where some personal data was not provided were not excluded but were not included in the analysis of that data. Geographical data of participants was not collected.

### 2.2 Statistical Analysis

All data was analysed using R version 4.2.2. A Shapiro–Wilk normality test was run on both diagnosis anxiety and treatment anxiety against all other variables (education, scenario presented, health insurance, previous pets with cancer and history of cancer personally or within the immediate family). Once the normality of the data was determined, a Levene test was run for homogeneity of variance: on diagnosis anxiety against all variables and on treatment anxiety against all variables, demonstrating equal variance among the groups.

A Kruskal–Wallis test was first used to determine the significance (*p* < 0.05) for the diagnosis anxiety level between the three terms addressed in the study: PTC, TPL and AC. When a statistically significant result was attained, a Wilcoxon rank sum with continuity correction was run between each pairings of the three groups (PTC vs. TPL, PTC vs. AC and TPL vs. AC). These tests were repeated for analysis of both treatment anxiety and determining the first‐choice treatment preference, with significance determined using a Kruskal–Wallis Test before moving onto a Wilcoxon rank sum with continuity correction. Anxiety scores for both diagnosis and treatment anxiety were assigned a numerical value from 1 to 5 to facilitate the running of these statistical tests, with one corresponding with ‘not at all anxious’ and five corresponding with ‘very anxious.’ The mean, variance and standard deviation were run on participants' ages and both treatment and diagnosis anxiety levels.

A MANOVA was run on diagnosis anxiety against education level, scenario, whether respondents had health insurance for their pets, whether respondents had a pet previously diagnosed with cancer and whether respondents had themselves been diagnosed or had a family member diagnosed with cancer previously. Treatments were assigned numerical values to facilitate the MANOVA. Tukey's Honestly Significant Difference test was run on the MANOVA subsequently. The same procedure was performed for treatment anxiety and, separately, treatment choice, with a MANOVA run and assessed secondarily by a Tukey's Honestly Significant Difference test for each.

Total responses for treatment choices were tallied to calculate the most selected and least selected treatments across scenarios; similarly, responses were tallied to determine which treatment choice was most commonly selected as one respondent would not choose at all in their selections.

## 3 Results

### 3.1 Respondent Demographics

To determine which scenario respondents would be shown, respondents were asked which third of the year they were born to randomise the terminology scenario displayed; those born January through April (*n* = 246, 36.02%) were given the diagnosis of PTC, those born May through August (*n* = 212, 31.04%) were given the diagnosis of ACs and those born September through December (*n* = 225, 32.94%) were given the diagnosis of TPL.

We collected a total of 683 respondents. Respondent demographics are outlined in Table 2. Six hundred and twenty‐two (91.7%) of the 678 respondents who provided gender data were female. The diagnosis of PTC, ACs and TPL were provided to 246 (36.02%), 212 (31.04%) and 225 (32.94%) respondents, respectively.

**TABLE 2:** Respondent demographics.

Variable^a^	Number of respondents	Percentage of respondents in category
Gender (*n* = 678)		
Male	43	6.34%
Female	622	91.74%
In another way	2	0.29%
Prefer not to say	11	1.62%
Age (*n* = 674)		
18–25 years	31	4.60%
26–35 years	87	12.91%
36–45 years	101	14.99%
46–55 years	168	24.93%
56–65 years	184	27.30%
> 65 years	103	15.28%
Highest education level completed (*n* = 666)		
Up to and including GCSEs/national qualifications	113	16.97%
A‐level study, International Baccalaureate (IB) or Senior Phase (up to S6) in Scotland	81	12.16%
Trade certificate or college diploma	113	16.97%
Undergraduate degree	214	32.13%
Postgraduate qualification (e.g., PhD, MSc)	145	21.77%
Pet at home (*n* = 683)		
Yes	662	96.93%
No	21	3.07%
Health insurance for any pets (*n* = 683)		
Yes for all pets	356	52.12%
Yes for some pets	108	15.81%
No	219	32.06%
Thyroid lump diagnosis in current or previous pets (*n* = 683)		
Yes	33	4.83%
No	650	95.17%
Any pet ever been diagnosed with cancer (*n* = 683)		
Yes	311	45.53%
No	372	54.47%
Personal or family diagnosis of a thyroid lump (*n* = 683)		
Yes	126	18.44%
No	549	80.38%
Prefer not to say	8	1.17%
Personal or family diagnosis of cancer of any type (*n* = 683)		
Yes	472	69.11%
No	203	29.72%
Prefer not to say	8	1.17%

^a^ Respondents were able to decline to answer questions pertaining to gender, age, highest educational level completed, a personal or family history of thyroid lumps and a personal or family history of cancer diagnoses.

### 3.2 Respondents Are More Likely to Choose Surgery When the Diagnosis Includes the Term ‘Cancer’

**FIGURE 1:**
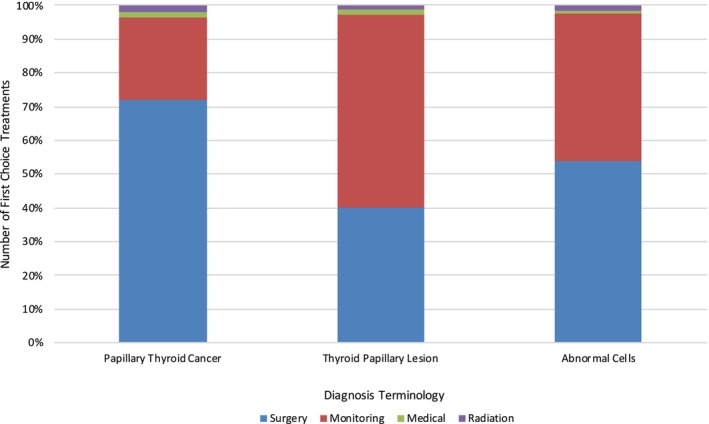
Number of first‐choice treatments by diagnosis terminology.

**FIGURE 2:**
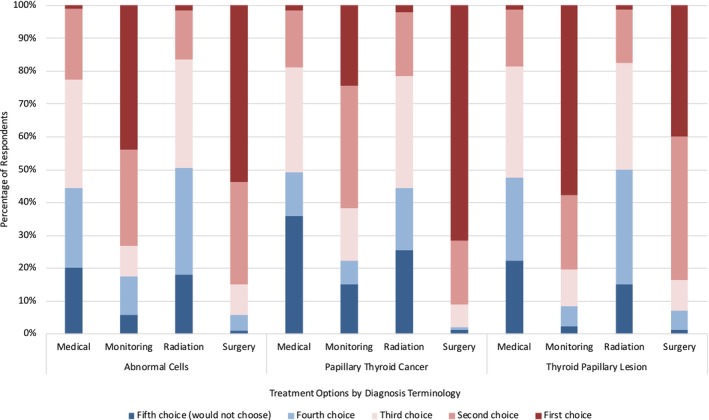
Treatment choice by diagnosis terminology.

We identified a significant difference in first‐choice treatment selection (*p* < 0.01) (Figure 1). Regardless of the diagnosis provided, we found that surgery was the most frequently chosen treatment, both as a first choice (55.8%, *n* = 380) and as a second to fourth choice of treatment (43.3%, *n* = 296) (Figure 2). The least frequently chosen treatment was radiation therapy, with just 1.18% of respondents selecting this as a first choice and 13.6% as a second to fourth choice of treatment. Irrespective of diagnosis, the treatment that respondents would not select as an option for their pet was most commonly medical therapy (53.2% of respondents), and the treatment choice that respondents selected the least in this category was surgery (2.35%). The order of most to least selected treatment options across all diagnoses was as follows: surgery, monitoring (active surveillance), medical therapy and radiation.

We found a significant difference in treatment choice based on the diagnosis terminology presented (*p* < 0.01), as well as a significant difference in treatment choice based on a previous experience of cancer in a pet (*p* < 0.02) The most significant difference in treatment choice based on diagnosis terminology came within the comparison of terms PTC and TPL (*p* < 0.01), whose respondents chose surgery as a first choice 71.8% and 39.8% of the time, respectively. This was followed by PTC and AC (*p* < 0.02) and TPL and AC (*p* < 0.04); those respondents shown the diagnosis of AC chose surgery as a first choice 53.8% of the time. Significant differences also appeared in treatment choices based on health insurance purchased for all pets versus health insurance for none or some pets (*p* = 0.05).

### 3.3 Respondents Are More Anxious About Diagnosis When the Term ‘Cancer’ Is Used

**FIGURE 3:**
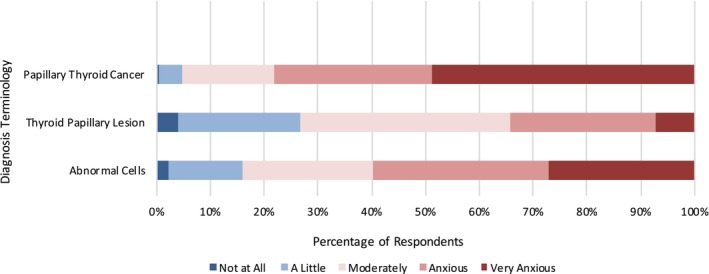
Level of diagnosis anxiety by diagnosis terminology.

We unveiled a significant difference in diagnosis anxiety levels depending on which diagnosis terms are used. When presented with a diagnosis of PTC, 78.1% of respondents reported being anxious or very anxious, compared with those who were assigned a diagnosis of TPL (34.2%) or ACs (59.4%) (*p* < 0.01) (Figure 3).

**FIGURE 4:**
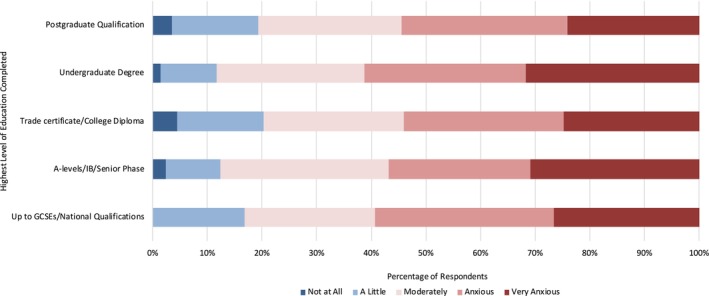
Diagnosis anxiety level by highest level of education completed.

We were interested in identifying relationships between respondent demographics and diagnosis anxiety. Using a MANOVA statistical analysis, we found a significant difference in diagnosis anxiety by education level (*p* < 0.04) and determined that the most substantial differences arose between respondents with undergraduate degrees vs. those with trade certificates, followed by those with undergraduate degrees vs. GSCEs and finally by those with postgraduate qualifications vs. those with trade certificates. Those with undergraduate degrees had the highest overall anxiety levels, with 61.2% of responses in the highest two anxiety brackets of ‘Anxious’ and ‘Very Anxious’, as compared with those respondents who had trade certificates (just 54% of responses in the highest two anxiety brackets) (Figure 4).

Interestingly, we also identified higher diagnosis anxiety in respondents who had pet health insurance policies compared with those who insured none of their pets (*p* = 0.05, MD 0.20). No other demographic variable was associated with the diagnosis of anxiety.

### 3.4 Respondents Are More Anxious About Treatment When the Term ‘Cancer’ Is Used

**FIGURE 5:**
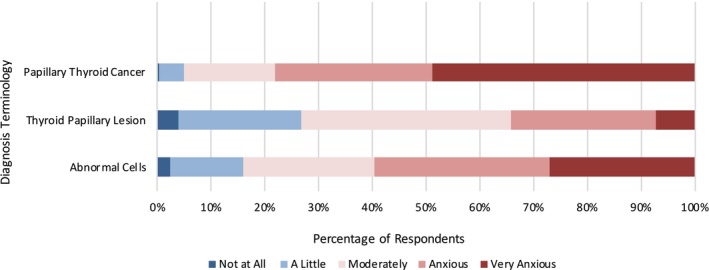
Level of treatment anxiety by diagnosis terminology.

We found that treatment anxiety closely mirrored diagnosis anxiety, with the diagnosis of PTC resulting in a greater treatment anxiety when compared with TPL (*p* < 0.01) and AC (*p* < 0.01). Additionally, a diagnosis of TPL was associated with a reported increase in treatment anxiety when compared with AC (*p* = 0.01) (Figure 5).

**FIGURE 6:**
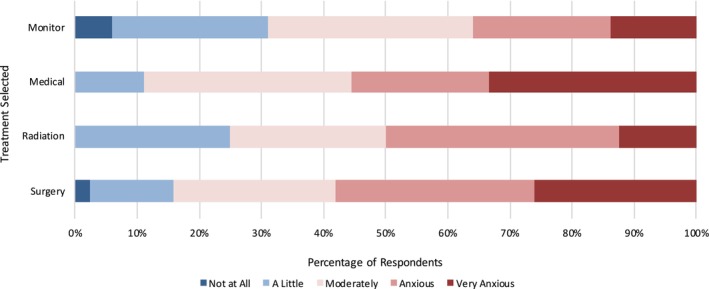
Level of anxiety by treatment selected.

Surgery was the treatment option most frequently selected, despite the highest levels of treatment anxiety being associated with this choice (Figure 6).

We determined that respondents' treatment anxiety was significantly greater in those with pet health insurance compared to those without pet health insurance (*p* < 0.01, MD 0.34). Additionally, a personal or immediate family experience of cancer was associated with a significantly increased treatment anxiety compared to respondents without this experience (*p* = 0.024). No other demographic variable was associated with treatment anxiety.

## 4 Discussion

To the authors' knowledge, there have been no studies addressing pet caregivers' or veterinarians' perception of different terminologies in veterinary oncology. In this study, we demonstrate that the terminology used to describe PTC may be associated with caregiver selection of treatment and levels of anxiety. We found that when the term ‘cancer’ was used, canine caregivers were more likely to select surgery as a treatment option for their pet. The term ‘cancer’ is also more likely to be associated with high levels of diagnosis and treatment anxiety compared with the terms ‘lesion’ and ‘abnormal cells’. Our findings mirror those in human medicine in theoretical scenarios for both thyroid cancers and mammary ductal carcinoma in situ; in Nickel et al. [20], the authors found that a diagnosis of PTC led patients to choose surgery far more than other therapies and experience significantly higher levels of anxiety surrounding this diagnosis. In studies for ductal carcinoma in situ, participants felt more anxious by diagnosis using the term ‘cancer’ than those without, and when ‘cancer’ was used in the diagnosis, patients opted for more aggressive treatments such as mastectomies or lumpectomies over active monitoring [23, 28].

Fattouh et al. [29] found that in human healthcare, higher levels of anxiety were associated with higher levels of education in patients with chronic disease; increased diagnosis anxiety in respondents with higher education levels in our study may suggest a greater understanding of the risks associated with the various treatment options presented or the diagnostic terms themselves. Increased treatment anxiety in those with a personal or family history of cancer has been found in several studies in human medicine and may similarly reflect a greater understanding of the disease process and the lived realities of any treatment choice [30, 31]. Higher diagnosis and treatment anxieties for owners who have taken out pet insurance policies for all pets may indicate owners who have or would want to exhaust more extensive treatment options for their own pets or may simply be more risk‐averse. As Williams et al. [32] found, owners with pet insurance spent more per veterinary visit than those without and that owners with pet insurance were more likely to opt for treatment over euthanasia.

This study faces many limitations, including its theoretical (scenario‐based) framework. Receiving diagnoses in person results in additional variables to consider, including the surgeon's influence in presenting the diagnosis on a pet owner's anxiety level and treatment preferences due to their manner of presenting information, experience level, age, gender and background. However, by sampling pet owners who had previous experience with cancers in pets and within their immediate family as well as those who were naïve to previous diagnoses of cancer, the section of the population sampled more accurately reflected clients coming to a veterinary clinic for a diagnosis of a thyroid lump. Results were biased towards those who join pet ownership groups online and by those who respond to studies in their entirety online; the female demographic was heavily over‐represented which may reflect the demographics of those in such groups. Ideally, an unbiased cross‐section of the population would be taken to include an equal distribution of gender, socioeconomic status, educational level, age and other participant characteristics. In addition, geographical data was not collected, and the high proportion of respondents reporting that their pet was insured may reflect either a higher proportion of respondents from countries where insurance is commonplace, or a tendency for those who insure their pets to be more likely to participate in surveys of this type.

Increasing access to diagnostic technology in the veterinary industry in recent years has massively expanded the ability of practitioners to diagnose thyroid masses quickly and accurately and to offer higher‐level treatment such as radiation therapy or bilateral thyroidectomies to clients [33, 34]. While PTC is rare in dogs, and overtreatment of canine thyroid tumours is unlikely, undertreatment of more aggressive cancers may be a risk depending on the terminology used. Advanced imaging modalities, a growing number of referral‐level veterinary hospitals, and additional diagnostic laboratories to send samples have all made the diagnosis of many cancers easier, providing owners with a greater number of treatment decisions and making discussions regarding the way these diagnoses and options are presented more topical than ever [35, 36, 37].

As Pieterse et al. [38] found that physician preference leads patients to prefer the physician's treatment choice over others available. It is critical, as the landscape of the healthcare professions changes, that medical staff are intentional in the way they present diagnosis and treatment options to prevent unintentionally biasing clients towards one treatment option over another, especially when diagnosing cancers with lower rates of metastasis, morbidity and mortality. Physicians must also avoid personal biases or assumptions of the client at the point of diagnosis and presentation of treatment options, suggesting choices they think clients will want to pursue based on patient background, socioeconomic status and other stereotypic factors [39]. Future research should investigate whether the findings from this paper—that the term ‘cancer’ greatly increases anxiety at both the point of diagnosis and in selecting treatment options and encourages immediate selection of more invasive treatment options like surgery—are applicable over a range of cancer diagnoses in veterinary medicine, especially for those that are benign or slowly progressive with low metastatic rates, which may make such treatments unnecessary. The researchers hypothesise that similar trends would be seen with a diagnosis using the term ‘cancer’ across tumour types given this trend in human medicine and would encourage veterinary medical staff to demonstrate consideration of terminology when providing such diagnoses until research demonstrates otherwise. Conversely, avoidance of the term ‘cancer’ may result in the under treatment of tumours, and surgical treatment is the most important potentially curative treatment for the majority of veterinary cancer patients. Interestingly, work investigating changing the terminology for low‐risk thyroid cancer in humans identified public concerns that changing terminology might risk disease progression because patients were less likely to seek treatment or to take their diagnoses seriously [40]. Clearly, there is much work to be done to ensure clear communication of tumour diagnoses and to optimise terminology, with associated education of human and veterinary healthcare professionals and the public.

## 5 Conclusion

In this study, we demonstrate that providing a diagnosis of *cancer* matters. The use of the term *cancer* is associated with caregiver anxiety and the choice of more invasive therapies. Clinicians who care for oncological patients should ensure that the diagnosis is described accurately and consistently and continue to advocate for the best management of individual cases, bearing in mind the impact of the terminology they use.

## Ethics Statement

This manuscript complies with all policies and ethical considerations of this journal. The study design was reviewed and approved by the University of Edinburgh Human Ethical Review Committee (HERC_2022_009).

## Conflicts of Interest

The authors no conflicts of interest.

## Supporting information


Appendix S1.

## Data Availability

The data that support the findings of this study are available on request from the corresponding author. The data are not publicly available due to privacy or ethical restrictions.
