# Supplementary material for: Effect of Changes in Canine Thyroid Cancer Terminology on Caregiver Anxiety Levels and Treatment Preferences in a Scenario‐Based Study
Source: Vet Comp Oncol. 2025 Feb 24;23(2):187–96. doi: 10.1111/vco.13044 (PMC12082792; doi:10.1111/vco.13044)
Supplement: Supplementary file 1 — Appendix S1. [file VCO-23-187-s001.docx]

Does the choice of words by vets influence owner's anxiety levels and treatment preferences

# Client Consent Form

This survey is part of a research project being carried out through the University of Edinburgh’s Royal (Dick) School of Veterinary Studies. At the Hospital for Small Animals, we wish to investigate whether the words we use to describe conditions seen in dogs has any influence on their family's decisions regarding treatment.

The survey should take no more than 15 minutes to complete.

Participation in the survey is voluntary. You may choose not to participate and you may withdraw at any time during the survey until you press the final submit button when your answers will be saved. As participation is anonymous, we will not be able to remove responses from the survey after you have submitted the data.

Your responses will be confidential. We do not collect identifying information such as your name, your email, or your IP address. Any data collected here will remain completely anonymous and will be stored on a secure folder within the University of Edinburgh server for one year, at which point it will be destroyed.

This project has received ethical approval from The Royal (Dick) School of Veterinary Studies Human Ethical Review Committee. The supervisor in charge of this project is Dr. Kelly Bowlt Blacklock of the University of Edinburgh. If you have any questions or concerns, please contact [kelly.blacklock@ed.ac.uk.](mailto:kelly.blacklock@ed.ac.uk)

By continuing with this survey, you are agreeing to the following conditions:

You have read the above information. You voluntarily agree to participate.

You are at least 18 years of age.

For any questions regarding the obligations of the researchers running this study under the General Data Protection Regulation (GDPR) 2016/679, please

see: https://[www.ed.ac.uk/data-protection/data-protection-policy](http://www.ed.ac.uk/data-protection/data-protection-policy)

I consent to participate


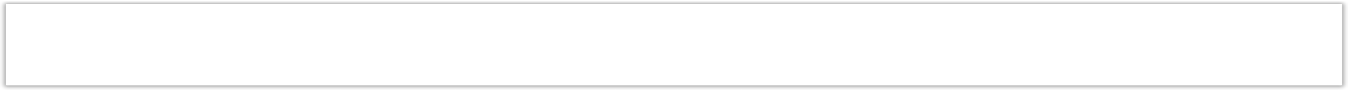

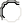


Yes

# Introduction

The thyroid is a small organ in the neck of every mammal (including dogs and humans) and it helps control growth and how the body uses energy.

In the following 3 fictional scenarios, please imagine that you own a dog with a condition affecting his/her thyroid. In each (seperate) scenario, the diagnosis is different but the 4 treatment options you are presented with are the same. We are interested in:

1. Which of the 4 treatments you would prefer for your dog in each scenario. There are no right or wrong answers.
2. We are also looking at the anxiety that arises from different terms used to describe disease of the thyroid in dogs. As such, this study involves asking you to reflect upon how anxious the diagnosis in the scenario makes you feel. We hope to use the information from this study to guide the terms we use in the future.

Treatment options include:

**Surgery:** Removing the entire thyroid gland by surgery, which is potentially curative. With surgery, there is a very small risk of dying from the surgery: less than 1.9%. Complications following surgery are seen in just under 13% of dogs and include bleeding (7.7% of dogs) and pneumonia (3.2% of dogs). Following surgery, there is a very small risk of low thyroid levels which necessitates lifelong medication (one tablet 1-2x daily).

**Monitoring (active surveillance):** Lifelong monitoring of the thyroid for growth or spread.

**Medical therapy (e.g. chemotherapy):** Chemotherapy is provided via a series of weekly treatments into the patient's vein, and has not classically been used in the treatment of thyroid cancer. Of dogs treated with chemotherapy and depending on the drug used, 20-50% will show some response. However, because chemotherapy is not commonly used, its role in extending survival time has not been

evaluated. Indications for chemotherapy may include cancer which is recurrent, which has spread or which is extremely large and not amenable to surgery.

Potential complications should be minimal, but include vomiting/diarrhoea and an increased risk of developing infections.

**Radiation therapy:** Radiation is more commonly indicated for very large thyroid tumours which are not amenable to surgery. Treatment consists of 4 weekly

treatments and mean survival time is around 45 months. Potential side effects are generally reversible and include hair loss and skin irritation over the neck.

Permanent side effects include change in hair and skin colour over the radiation site.

# Birth month

In which of the following months were you born?  *Required*


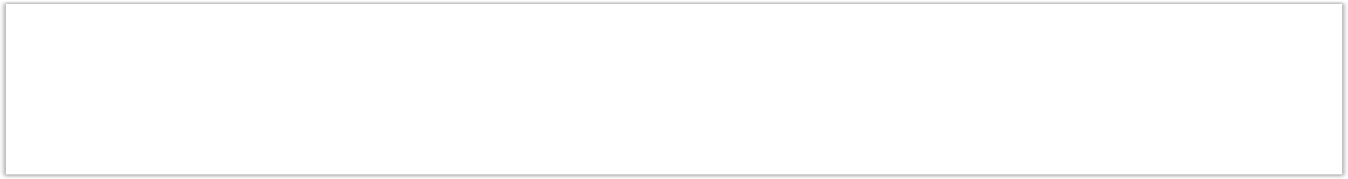

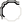

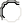

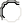


January, February, March, or April May, June, July, or August

September, October, November, or December

# Scenario 1

You were petting your dog at home when you found a 1 cm lump in his/her throat. You have not noticed any other problems with your dog and the lump does not seem to be bothering him.

You took your dog to your veterinary surgeon, where the vet told you that the lump was on your dog’s thyroid gland, and recommends a small needle biopsy of the lump.

The veterinary surgeon tells you the biopsy shows the lump to be a **papillary thyroid cancer**.

How anxious does this diagnosis make you feel?  *Required*


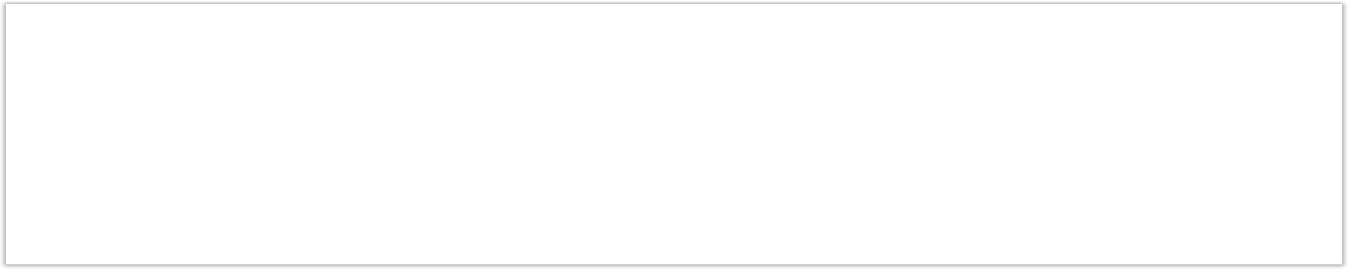

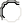

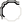

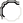

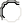

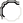


Not at all anxious A little anxious

Moderately anxious Anxious

Very anxious

**Treatment options include:**

**Surgery:** Removing the entire thyroid gland by surgery, which is potentially curative. With surgery, there is a very small risk of dying from the surgery: less than 1.9%. Complications following surgery are seen in just under 13% of dogs and include bleeding (7.7% of dogs) and pneumonia (3.2% of dogs). Following surgery, there is a very small risk of low thyroid levels which necessitates lifelong medication (one tablet 1-2x daily).

**Monitoring (active surveillance):** Lifelong monitoring of the thyroid for growth or spread.

**Medical therapy (e.g. chemotherapy):** Chemotherapy is provided via a series of weekly treatments into the patient's vein, and has not classically been used in the treatment of thyroid cancer. Of dogs treated with chemotherapy and depending on the drug used, 20-50% will show some response. However, because chemotherapy

is not commonly used, its role in extending survival time has not been evaluated. Indications for chemotherapy may include cancer which is recurrent, which has spread or which is extremely large and not amenable to surgery.

Potential complications should be minimal, but include vomiting/diarrhoea and an increased risk of developing infections.

**Radiation therapy:** Radiation is more commonly indicated for very large thyroid tumours which are not amenable to surgery. Treatment consists of 4 weekly treatments and mean survival time is around 45 months. Potential side effects are generally reversible and include hair loss and skin irritation over the neck.

Permanent side effects include change in hair and skin colour over the radiation site.

Please select which of these treatment options would be your preferred 1st, 2nd, 3rd or 4th choice following a diagnosis of papillary thyroid cancer in your pet (assuming all options are available locally and your pet is fully insured). There are no right or wrong answers, we just want to know what treatment you prefer.

|  | Surgery | Monitoring (active surveillance) | Medical therapy | Radiation therapy |
| --- | --- | --- | --- | --- |
| 1st Choice |  |  |  |  |
| 2nd Choice |  |  |  |  |
| 3rd Choice |  |  |  |  |
| I would not choose this treatment |  |  |  |  |

In thinking about the treatment option you have just selected to treat **papillary thyroid cancer** in your dog, how anxious does that treatment option make you feel?  *Required*


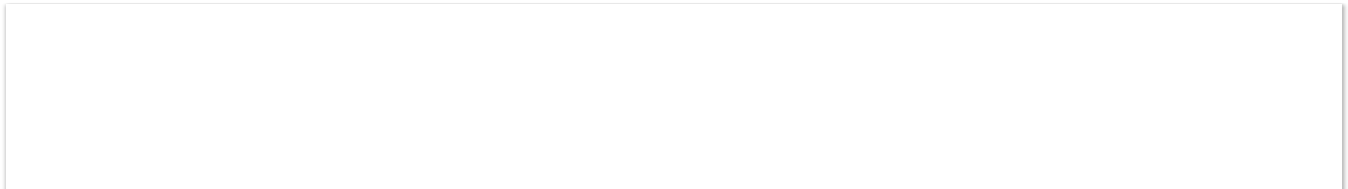

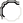

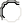

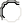

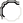


Not at all anxious A little anxious

Moderately anxious

Anxious


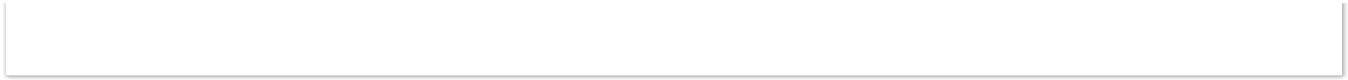

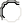


Very anxious

# Scenario 2

You were petting your dog at home when you found a 1 cm lump in his/her throat. You have not noticed any other problems with your dog and the lump does not seem to be bothering him.

You took your dog to your veterinary surgeon, where the vet told you that the lump was on your dog’s thyroid gland, and recommends a small needle biopsy of the lump.

The veterinary surgeon tells you the biopsy shows the lump to be a **thyroid papillary lesion.**

How anxious does this diagnosis make you feel?  *Required*


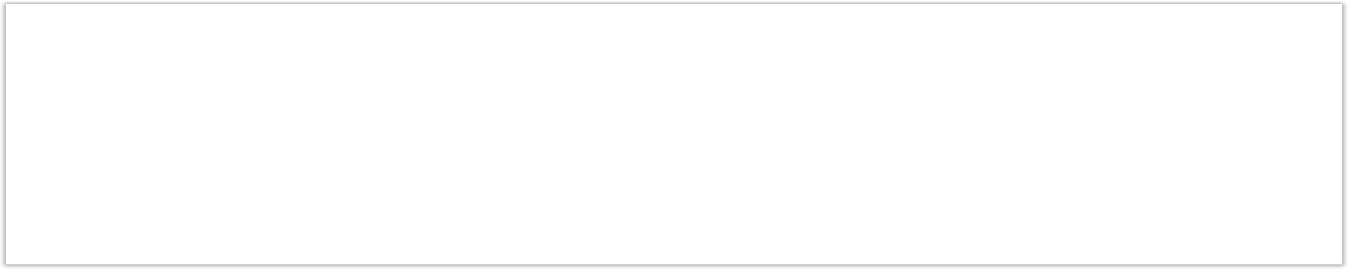

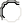

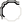

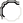

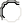

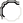


Not at all anxious A little anxious

Moderately anxious Anxious

Very anxious

**Treatment options include:**

**Surgery:** Removing the entire thyroid gland by surgery, which is potentially curative. With surgery, there is a very small risk of dying from the surgery: less than 1.9%. Complications following surgery are seen in just under 13% of dogs and include bleeding (7.7% of dogs) and pneumonia (3.2% of dogs). Following surgery, there is a very small risk of low thyroid levels which necessitates lifelong medication (one tablet 1-2x daily).

**Monitoring (active surveillance):** Lifelong monitoring of the thyroid for growth or spread.

**Medical therapy (e.g. chemotherapy):** Chemotherapy is provided via a series of weekly treatments into the patient's vein, and has not classically been used in the treatment of thyroid cancer. Of dogs treated with chemotherapy and depending on

the drug used, 20-50% will show some response. However, because chemotherapy is not commonly used, its role in extending survival time has not been

evaluated. Indications for chemotherapy may include cancer which is recurrent, which has spread or which is extremely large and not amenable to surgery.

Potential complications should be minimal, but include vomiting/diarrhoea and an increased risk of developing infections.

**Radiation therapy:** Radiation is more commonly indicated for very large thyroid tumours which are not amenable to surgery. Treatment consists of 4 weekly treatments and mean survival time is around 45 months. Potential side effects are generally reversible and include hair loss and skin irritation over the neck.

Permanent side effects include change in hair and skin colour over the radiation site.

Please select which of these treatment options would be your preferred 1st, 2nd, 3rd or 4th choice following a diagnosis of thyroid papillary lesion in your pet (assuming all options are available locally and your pet is fully insured). There are no right or wrong answers, we just want to know what treatment you prefer.

|  | Medical therapy | Monitoring (active surveillance) | Surgery | Radiation therapy |
| --- | --- | --- | --- | --- |
| 1st Choice |  |  |  |  |
| 2nd Choice |  |  |  |  |
| 3rd Choice |  |  |  |  |
| 4th Choice |  |  |  |  |
| I would not choose this treatment option |  |  |  |  |

In thinking about the treatment option you have just selected to treat **thyroid papillary lesion** in your dog, how anxious does that treatment option make you feel?  *Required*


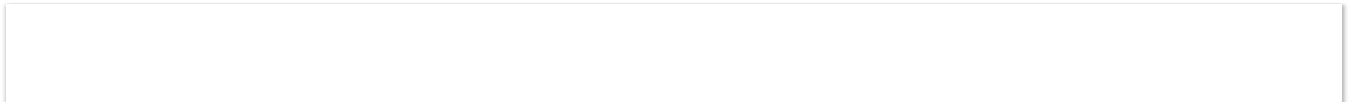

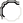

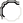


Not at all anxious A little anxious


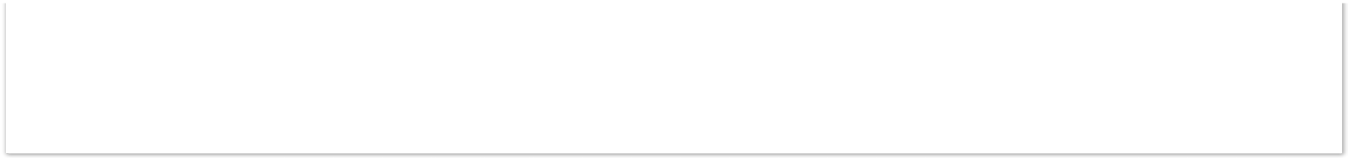

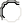

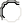

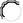


Moderately anxious Anxious

Very anxious

# Scenario 3

You were petting your dog at home when you found a 1 cm lump in his/her throat. You have not noticed any other problems with your dog and the lump does not seem to be bothering him.

You took your dog to your veterinary surgeon, where the vet told you that the lump was on your dog’s thyroid gland, and recommends a small needle biopsy of the lump.

The veterinary surgeon tells you the biopsy shows the lump to be **abnormal cells**.

How anxious does this diagnosis make you feel?  *Required*


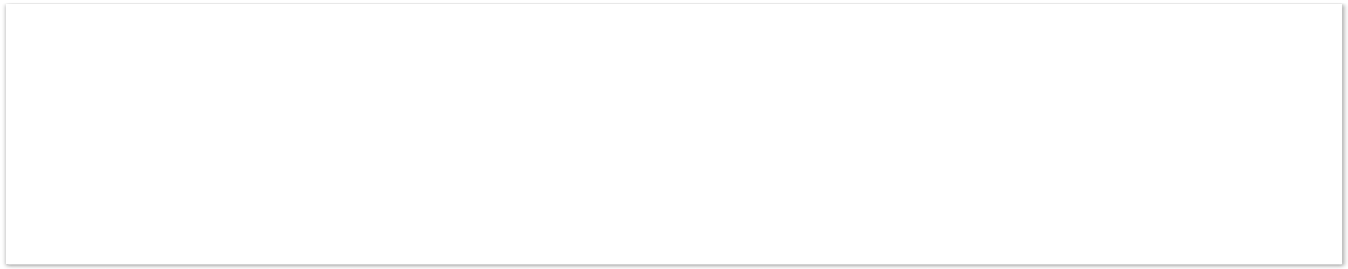

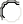

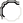

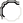

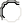

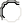


Not at all anxious A little anxious

Moderately anxious Anxious

Very anxious

**Treatment options include:**

**Surgery:** Removing the entire thyroid gland by surgery, which is potentially curative. With surgery, there is a very small risk of dying from the surgery: less than 1.9%. Complications following surgery are seen in just under 13% of dogs and include bleeding (7.7% of dogs) and pneumonia (3.2% of dogs). Following surgery, there is a very small risk of low thyroid levels which necessitates lifelong medication (one tablet 1-2x daily).

**Monitoring (active surveillance):** Lifelong monitoring of the thyroid for growth or spread.

**Medical therapy (e.g. chemotherapy):** Chemotherapy is provided via a series of weekly treatments into the patient's vein, and has not classically been used in the treatment of thyroid cancer. Of dogs treated with chemotherapy and depending on the drug used, 20-50% will show some response. However, because chemotherapy is not commonly used, its role in extending survival time has not been

evaluated. Indications for chemotherapy may include cancer which is recurrent, which has spread or which is extremely large and not amenable to surgery.

Potential complications should be minimal, but include vomiting/diarrhoea and an increased risk of developing infections.

**Radiation therapy:** Radiation is more commonly indicated for very large thyroid tumours which are not amenable to surgery. Treatment consists of 4 weekly treatments and mean survival time is around 45 months. Potential side effects are generally reversible and include hair loss and skin irritation over the neck.

Permanent side effects include change in hair and skin colour over the radiation site.

Please select which of these treatment options would be your preferred1st, 2nd, 3rd or 4th option following a diagnosis of abnormal cells in your pet (assuming all options are available locally and your pet is fully insured). There are no right or wrong answers, we just want to know what treatment you prefer.

|  | Monitoring (active surveillance) | Medical therapy | Radiation | Surgery |
| --- | --- | --- | --- | --- |
| 1st Choice |  |  |  |  |
| 2nd Choice |  |  |  |  |
| 3rd Choice |  |  |  |  |
| 4th Choice |  |  |  |  |
| I would not choose this treatment option |  |  |  |  |

In thinking about the treatment option you have just selected to treat **abnormal cells** in your dog, how anxious does that treatment option make you feel?  *Required*


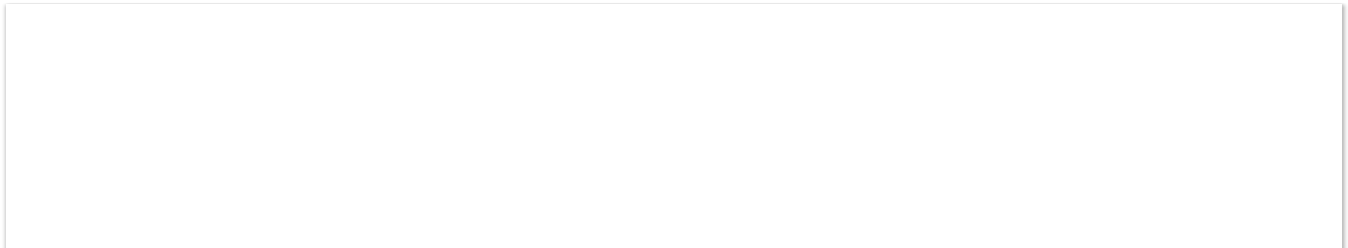

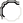

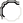

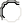

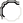

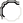


Not at all anxious A little anxious

Moderately anxious Anxious

Very anxious


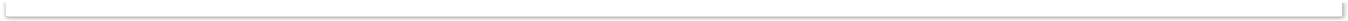


# Background Information on the Respondent

With what gender do you identify? *Optional*


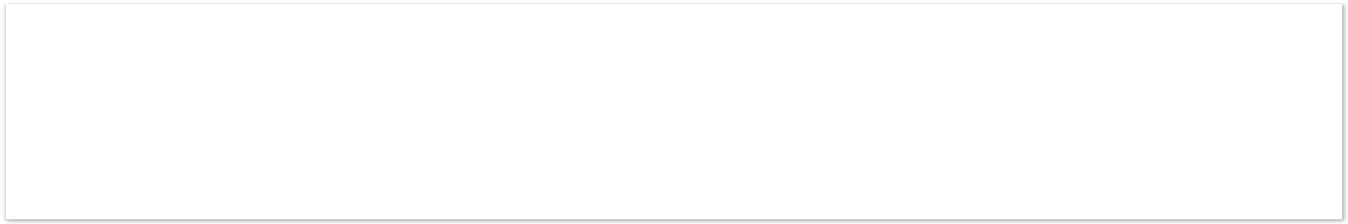

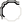

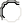

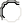

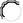


Male Female

In another way Prefer not to say

How old are you? *Optional*


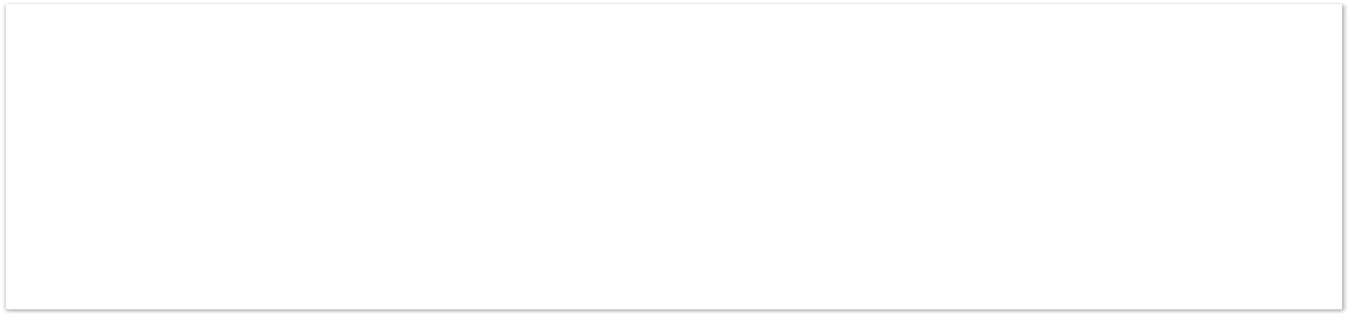

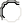

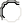

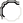

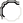

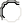

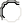


18-25 years old

26-35 years old

36-45 years old

46-55 years old

56-65 years old Over 65 years old

What is your highest educational level completed? *Optional*


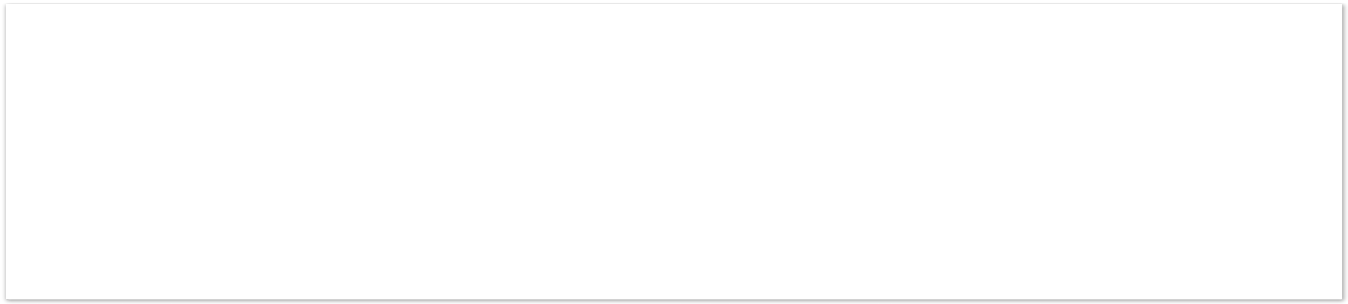

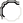

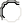

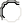

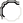

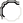


Up to and including GCSEs/National Qualifications

A-level study, International Baccalaureate (IB), or Senior Phase (up to S6) in Scotland

Trade certificate or college diploma Undergraduate degree

Postgraduate qualification (e.g. PhD, MSc)

Do you currently have a pet at home?  *Required*


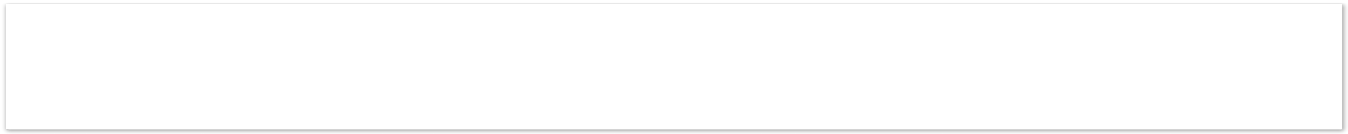

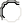

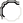


Yes No

Do you have health insurance plans for any of your pets?  *Required*


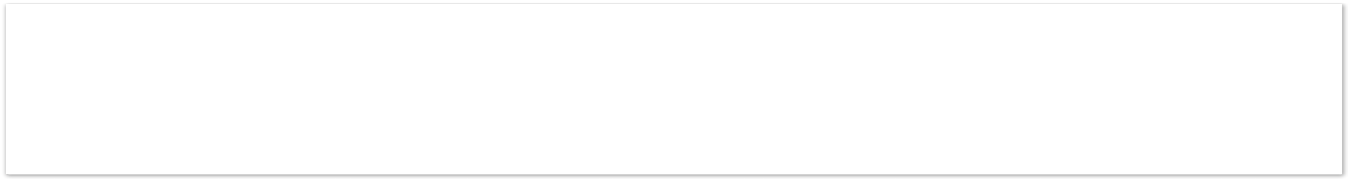

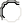

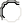

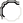


Yes, for all of my pets

Yes, but only for some of my pets No

Has a thyroid lump been diagnosed in any of your current or previous pets?  *Required*


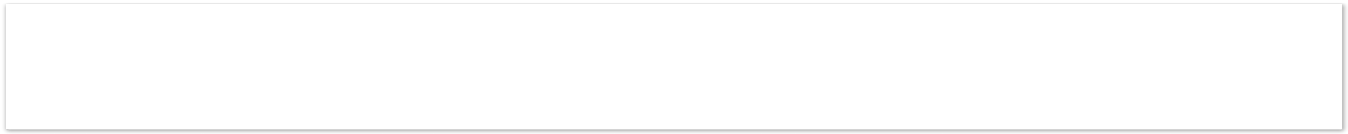

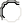

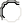


Yes No

Has a pet of yours ever received a diagnosis of cancer?  *Required*


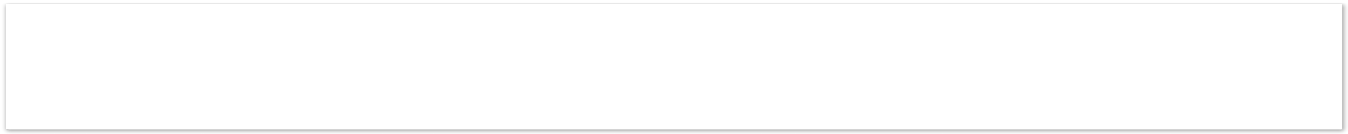

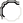

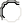


Yes No

Have you or anyone in your immediately family had a thyroid lump? *Optional*


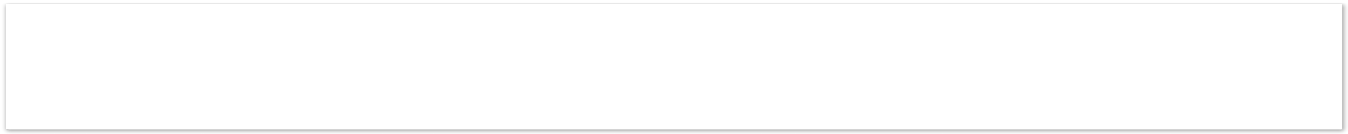

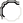

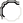


Yes No

Have you or anyone in your immediate family received a diagnosis of cancer of any type? *Optional*


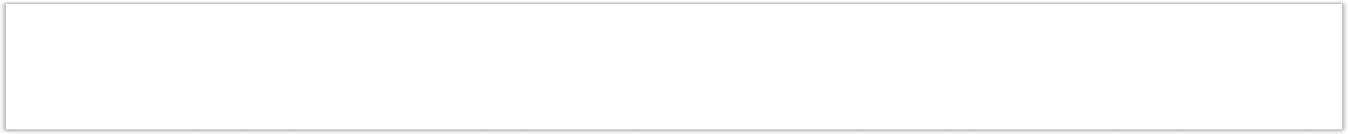

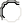

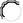


Yes No

Final page

Thank you so much for participating in this survey. If you have any questions, comments, or concerns with this survey, please contact Dr. Kelly Bowlt Blacklock at [kelly.blacklock@ed.ac.uk.](mailto:kelly.blacklock@ed.ac.uk)
